# A Return to the Logic-First Spirit of Corey’s Retrosynthetic Analysis, Now Implemented in Modern Data-Driven CASP

**DOI:** 10.1021/acscentsci.6c00702

**Published:** 2026-04-29

**Authors:** Mikhail Kabeshov, Giulia Bergonzini, Ola Engkvist

**Affiliations:** † Molecular AI, Discovery Sciences, R&D, AstraZeneca Gothenburg, Pepparedsleden 1, Mölndal 43183, Sweden; ‡ Compound Synthesis and Management, Discovery Sciences, R&D, AstraZeneca Gothenburg, Pepparedsleden 1, Mölndal 43183, Sweden

## Abstract

A higher-level retrosynthesis strategy helps computers search synthesis space more like chemists do, improving route discovery for challenging molecular targets.

## Graphical abstract

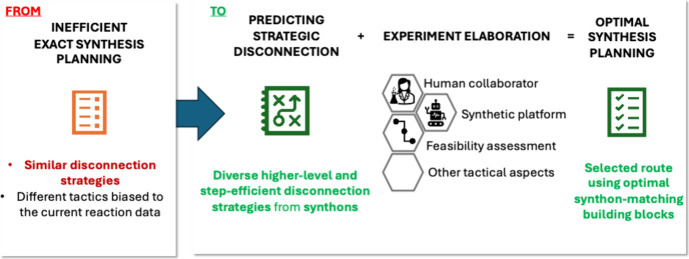

In a recent issue of *ACS Central Science*, Connor W. Coley and co-workers report a strikingly simple but important shift in how computers can plan organic syntheses: instead of committing too early to exact reagents, leaving groups, and protecting-group maneuvers, their algorithm first reasons at the level of higher-level strategy.^1^

Retrosynthesis has always been about reducing complexity. Chemists look at a target molecule and ask not only “What reaction should I do next?” but, more fundamentally, “What is the best way to disconnect this structure into meaningful fragments?” That distinction matters. Human experts often begin with broad strategic disconnections and only later decide which exact functional groups, reagents, or tactical adjustments will make those ideas work in the laboratory. Corey’s classic vision of retrosynthesis was built around this kind of logic-driven simplification.^2–4^

Modern computer-aided synthesis planning (CASP) has made enormous progress. Data-driven systems can now learn from very large reaction sets, propose plausible one-step disconnections, and search multistep routes far more effectively than earlier rule-only systems. Landmark advances include neural-network-guided route planning with Monte Carlo tree search,^5^ practical open-source platforms such as ASKCOS and AiZynthFinder,^6^ and transformer-based systems^7^ that broadened the range of learned retrosynthetic proposals.

And yet, there has remained a mismatch between how chemists think and how many algorithms search. Most current CASP systems plan at the level of fully specified reactions.^8–10^ That means the search can become crowded with proposals that are tactically different but strategically similar: one route may differ from another only by the precise leaving group, oxidation level adjustment, or protecting group choice. For easy targets that may be acceptable. For difficult targets, it can become a serious burden, because the search tree grows wider and deeper while the underlying strategic options do not really become more informative. The conceptual leap in this paper is to ask the computer to postpone tactical details and focus first on the disconnection logic that chemists care about most.

The conceptual leap in this paper is to ask the computer to postpone tactical details and focus first on the disconnection logic that chemists care about most.

Roh, Joung, Yu, Tu, Bartholomew, Santiago-Reyes, Fong, Sarpong, Reisman, and Coley do this by abstracting detailed substructures that make it into the final product—synthons. In practice, that means treating several functionally related reactants as the same synthon. A carboxylic acid derivative, for example, can be represented at the level of an acyl cation equivalent rather than forcing the planner to decide immediately whether the forward route should use an acid chloride, ester, or acid itself. The result is a planning representation that emphasizes scaffold-building chemistry and suppresses purely tactical interconversions until later. This is not chemistry denial; it is chemistry sequencing. Strategy comes first, implementation second. That framing feels naturally aligned with how experienced synthetic chemists actually work.

The practical outcome is compelling. The authors show that the higher-level representation reduces route complexity in benchmark data and improves the planner’s ability to find routes for more challenging targets. Just as importantly, the routes it proposes are often shorter in the abstract planning space because they do not waste effort on tactical steps that can be sensibly reintroduced later. That does not mean the laboratory synthesis will necessarily contain fewer operations in every case. It means the algorithm spends more of its computational effort on the decisions that define a synthesis rather than on bookkeeping around it. By collapsing tactically different but strategically equivalent reactions into common abstractions, the method makes route search more efficient without abandoning chemical intuition.

By collapsing tactically different but strategically equivalent reactions into common abstractions, the method makes route search more efficient without abandoning chemical intuition.

This is where the paper becomes especially interesting for practicing chemists. The authors do not claim that higher-level retrosynthesis is a complete replacement for detailed synthesis design. Rather, they position it as a better first stage. Once a promising strategic route is identified, chemists can map it back into concrete reagents, select protecting groups, choose among buyable building blocks, and adapt the plan to feasibility, selectivity, scale, or platform constraints. That human–AI handoff is one of the strongest features of the study. Instead of forcing chemists to accept a brittle, overcommitted route, the system provides a strategic scaffold on which expert judgment can operate.

The case studies make this tangible. For complex molecules such as narlaprevir and natural products including pinolidoxin and pendolmycin, the higher-level approach can recover routes that more closely reflect meaningful bond-forming logic, even where conventional planning struggles. The point is not merely that the computer finds “a route.” It is that it finds a route at a level that a synthetic chemist can recognize, critique, and build on. This work suggests that the next leap in computer-aided synthesis planning may come not from making algorithms specify more, but from teaching them when to specify less.

This work suggests that the next leap in computer-aided synthesis planning may come not from making algorithms specify more, but from teaching them when to specify less.

The broader implication is that retrosynthesis may benefit from being split into two linked problems. First, identify the strategic architecture of a route. Second, elaborate that architecture into executable chemistry using conditions prediction, feasibility assessment, and experimental constraints. That separation could prove valuable not only for human-guided planning but also for integration with automated synthesis platforms, where the choice of exact building blocks and tactical steps may depend on local inventory, robotics constraints, or fresh reaction data. This paper therefore feels important not only because it improves a benchmark, but because it recenters CASP on the organizing ideas of synthesis. In doing so, it brings computer planning closer to the original spirit of retrosynthetic analysis: simplify first, decide later, and let strategy guide tactics rather than the reverse. However, one should also note that as more progress is made in developing novel algorithms for synthetic route prediction, the more the quality of the available reaction data becomes the bottleneck for further progress in the field. This needs to be addressed through integration with automated experimental workflows.
